# 4-(3-Fluoro­phen­yl)-1-(propan-2-yl­idene)thio­semicarbazone

**DOI:** 10.1107/S1600536811042504

**Published:** 2011-10-22

**Authors:** Barbara Miroslaw, Daniel Szulczyk, Anna E. Koziol, Marta Struga

**Affiliations:** a Faculty of Chemistry, Maria Curie-Sklodowska University, pl. M. Curie-Sklodowskiej 3, 20-031 Lublin, Poland; b Department of Medical Chemistry, The Medical University, 3 Oczki Str., 02-007 Warsaw, Poland

## Abstract

The title compound, C_10_H_12_FN_3_S, crystallizes in the same space group (*P*2_1_/*c*) as two polymorphic forms of 4-phenyl-1-(propan-2-yl­idene)thio­semicarbazone [Jian *et al.* (2005). *Acta Cryst.* E**61**, o653–o654; Venkatraman *et al.* (2005). *Acta Cryst.* E**61**, o3914–o3916]. The arrangement of mol­ecules relative to the twofold screw axes is similar to that in the crystal structure of the lower density polymorph. In the solid state, the mol­ecular conformation is stabilized by an intra­molecular N—H⋯N hydrogen bond. The mol­ecules form centrosymmetric *R*_2_^2^(8) dimers in the crystal through pairs of N—H⋯S hydrogen bonds.

## Related literature

For related structures, see: Basu & Das (2011 ▶); Park & Ahn (1985 ▶); Parsons *et al.* (2000 ▶); Jian *et al.* (2005 ▶); Venkatraman *et al.* (2005 ▶). For description of the Cambridge Structural Database, see: Allen (2002 ▶). For the anti­tumor, anti­viral and anti­fungal activity of thio­semicarbazones, see: Kalinowski *et al.* (2009 ▶); Smee & Sidwell (2003 ▶); Beraldo & Gambino (2004 ▶). For their metal-chelating properties, see: Paterson & Donnelly (2011 ▶); Casas *et al.* (2000 ▶).
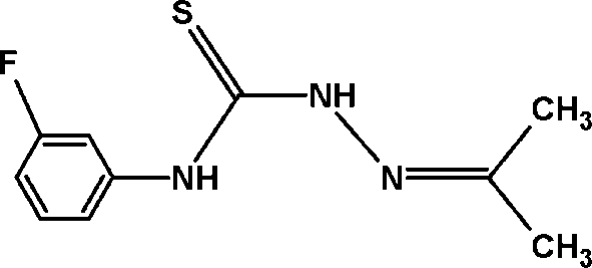


## Experimental

### 

#### Crystal data

C_10_H_12_FN_3_S
*M* *_r_* = 225.29
Monoclinic, 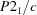

*a* = 9.038 (2) Å
*b* = 10.515 (2) Å
*c* = 11.869 (2) Å
β = 99.77 (3)°
*V* = 1111.6 (4) Å^3^
*Z* = 4
Cu *K*α radiation
μ = 2.48 mm^−1^
*T* = 296 K
0.55 × 0.30 × 0.10 mm

#### Data collection

Kuma KM-4 diffractometer
Absorption correction: for a cylinder mounted on the ϕ axis (Dwiggins, 1975 ▶) *T*_min_ = 0.435, *T*_max_ = 0.485
3800 measured reflections
1942 independent reflections
1252 reflections with *I* > 2σ(*I*)
*R*_int_ = 0.084
3 standard reflections every 100 reflections  intensity decay: 3.3%

#### Refinement

*R*[*F* ^2^ > 2σ(*F* ^2^)] = 0.068
*wR*(*F* ^2^) = 0.217
*S* = 1.04
1942 reflections
138 parameters
H-atom parameters constrained
Δρ_max_ = 0.38 e Å^−3^
Δρ_min_ = −0.39 e Å^−3^

### 

Data collection: *KM-4 Software* (Kuma Diffraction, 1991 ▶); cell refinement: *KM-4 Software*; data reduction: *KM-4 Software*; program(s) used to solve structure: *SHELXS97* (Sheldrick, 2008 ▶); program(s) used to refine structure: *SHELXL97* (Sheldrick, 2008 ▶); molecular graphics: *DIAMOND* (Brandenburg, 1999 ▶), *Mercury* (Macrae *et al.*, 2006 ▶) and *ORTEP-3* for Windows (Farrugia, 1997 ▶); software used to prepare material for publication: *SHELXL97*.

## Supplementary Material

Crystal structure: contains datablock(s) I, global. DOI: 10.1107/S1600536811042504/fy2024sup1.cif

Supplementary material file. DOI: 10.1107/S1600536811042504/fy2024Isup2.mol

Structure factors: contains datablock(s) I. DOI: 10.1107/S1600536811042504/fy2024Isup3.hkl

Supplementary material file. DOI: 10.1107/S1600536811042504/fy2024Isup4.cml

Additional supplementary materials:  crystallographic information; 3D view; checkCIF report

## Figures and Tables

**Table 1:** Hydrogen-bond geometry (Å, °)

*D*—H⋯*A*	*D*—H	H⋯*A*	*D*⋯*A*	*D*—H⋯*A*
N1—H1⋯N3	0.86	2.12	2.553 (5)	111
N2—H2⋯S1^i^	0.86	2.67	3.465 (3)	154

Symmetry code: (i) 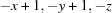.

**Fig. 1.**
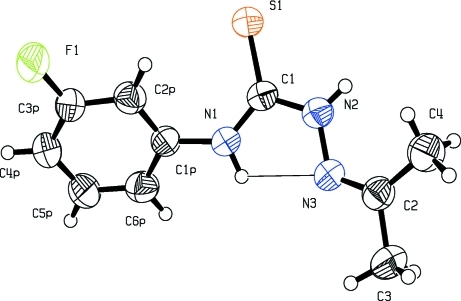
Molecular structure of the title compound with 50% probability displacement ellipsoids. H atoms are shown as small spheres of an arbitrary size. Thin single line represents the intramolecular hydrogen bond.

**Fig. 2.**
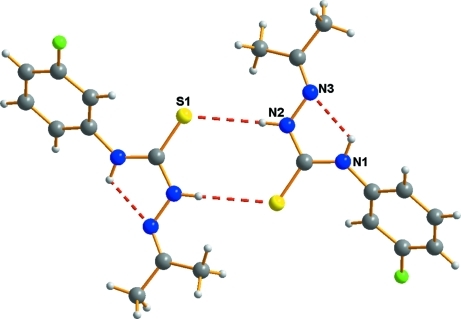
Dimer in (I) formed by hydrogen bonds around a centre of symmetry.

**Fig. 3.**
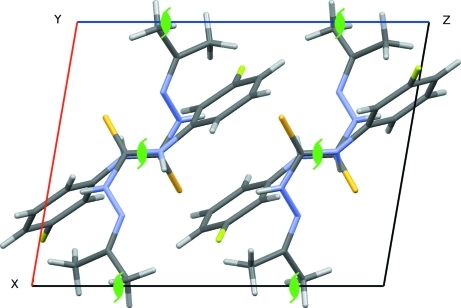
Orientation of molecules in relation to the 21 screw axes in (I). View along the b axis. Green symbols indicate the positions of 21 screw axes in the unit cells.

**Fig. 4.**
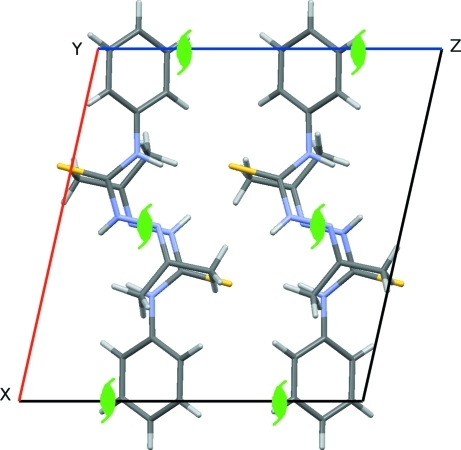
Orientation of molecules in relation to the 21 screw axes in FIDDUS01. View along b axis. Green symbols indicate positions of 21 screw axes in the unit cells.

## supplementary crystallographic information

### Comment

Thiosemicarbazones are widely studied due to their antitumor, antiviral and antifungal activity (Kalinowski *et al.*, 2009; Smee & Sidwell, 2003; Beraldo & Gambino, 2004) as well as for their metal chelating properties (Paterson & Donnelly, 2011; Casas *et al.*, 2000). The molecular structure of the title compound (I) with numbering scheme is shown in Fig. 1. Recently, two crystal forms of 4-phenyl-1-(propan-2-ylidene)thiosemicarbazone have been reported. However, they were not identified as polymorphs (Jian *et al.*, 2005, CSD *Refcode:* FIDDUS; Venkatraman *et al.*, 2005, *Refcode*: FIDDUS01). Crystals of FIDDUS were obtained by recrystallisation from dimethyl sulfoxide, while those of FIDDUS01 from an acetone:methanol solution. The crystal structure reported here is similar to the lower density polymorph, FIDDUS (*d* = 1.258 Mg/m^-3^*vs.* 1.302 Mg/m^-3^ for FIDDUS01).

The bond lengths confirm the thione form of the title molecule, the presence of a double bond between the N3 and C2 atoms, and they indicate some π-delocalisation along the thiosemicarbazone fragment. The S1 and the hydrazinic N3 atoms are in the *trans* conformation with the S1—C1—N2—N3 torsion angle equal to -177.7 (3)°. This conformation enables the formation of an intramolecular N1—H···N3 hydrogen bond. Consequently, the thiosemicarbazone part of the molecule (N1—C1—S1—N2—N3) is planar, with the maximum deviation from the mean plane of these atoms being 0.014 (2) Å for N3. This conformation seems to be characteristic of the thiosemicarbazone fragment, and it is observed in all related crystal structures found in the CSD (Allen, 2002) [*Refcodes*: CUZXOK (Parsons *et al.*, 2000), DAWPOG (Park & Ahn, 1985), FIDDUS (Jian *et al.*, 2005), FIDDUS01 (Venkatraman *et al.*, 2005) and UQOWAZ (Basu & Das, 2011))]. The dihedral angles between the central thiosemicarbazone plane of (I) and the planes formed by the propan-2-ylidene (C2—C3—C4) and phenyl (C1P to C6P) groups are 16.7 (4) and 38.9 (2)°, respectively. For comparison, the respective angles in FIDDUS are 14.0° and 38.6°; and in FIDDUS01 they are 23.6° and 42.8°.

The thiosemicarbazone part is also involved in intermolecular (N2—H···S1) hydrogen bonds (Fig. 2, Table 1), resulting in *R*^2^_2_(8) centrosymmetric dimers. (The same pattern have been found in DAWPOG, FIDDUS, FIDDUS01 and UQOWAZ.) The main difference between the two aforementioned polymorphs is the orientation of the molecules relative to the twofold screw axes. In FIDDUS and in (I) the 2_1 _screw axis passes through the C1—N1 bond, while in FIDDUS01 it runs through the N2—N3 bond (Figs. 3 and 4). Additionally, in FIDDUS01 the thiosemicarbazone plane is almost perpendicular to the *b* direction (85.7°) while in FIDDDUS and in (I) the corresponding angles are 62.0° and 59.9 (4)°, respectively. In (I) and in FIDDUS there are offset stacking interactions between the aromatic rings (Fig. 3) with interplanar distances of 3.5 (1) Å and 3.6 Å, respectively. The physical consequence of these stacking interactions is the yellow colour of FIDDUS crystals, in contrast to the colourless crystals of FIDDUS01, where overlapping of the heteroatoms is observed (Fig. 4). Surprisingly, the crystals of (I) are colourless, despite the similar molecular and crystal structure with FIDDUS. This could be explained by the changes in the electronic structure of the aromatic ring in molecule (I) caused by the electronegative fluorine substituent. The presence of the fluorine atom in (I) causes only slight differences in crystal packing with respect to FIDDUS. In (I) there is a short intermolecular contact between the F1 and C2 (1 - *x*, 1/2 + *y*, 0.5 - *z*) atoms with a distance of 3.107 (5) Å (the sum of van der Waals radii is 3.17 Å).

### Experimental

The title compound, C_10_H_12_FN_3_S, was obtained in the reaction of 4-amino-1,7,8,9,10-pentamethyl-4-azatricyclo[2.5.1.0^2,6^]dec-8-ene-3,5-dione and 3-fluorophenyl isothiocyanate in acetonitrile. The mixture of the reagents was refluxed for 6 h. After heating, the solvent was removed on a rotary evaporator. The residue was purified by column chromatography (chloroform:methanol 5.5:0.5). Two products were obtained in this reaction, *viz*.: 1-(3-fluorophenyl)-3-(1,7,8,9,10-pentametyl-3,5-dioxo-4-azatricyclo[5.2.1.0^2,6^]dec-8-en-4-yl)thiourea (60%) and 4-(3-fluorophenyl)-1-(propan-2-ylidene)thiosemicarbazone (40%). The title compound was recrystallised from acetonitrile.

### Refinement

All C-bonded H atoms were positioned geometrically and allowed to ride on the attached atom with C—H bond lengths of 0.93 Å for aromatic atoms and 0.96 Å for methyl groups. The positions of N-bonded H atoms were located in the difference electron density maps and then constrained with an N—H distance of 0.86 Å. *U*_iso_(H) values were fixed to 1.2*U*_eq_(C,N).

### Crystal data

**Table:**

C_10_H_12_FN_3_S	*F*(000) = 472
*M**_r_* = 225.29	*D*_x_ = 1.346 Mg m^−^^3^
Monoclinic, *P*2_1_/*c*	Cu *K*α radiation, λ = 1.54178 Å
Hall symbol: -P 2ybc	Cell parameters from 75 reflections
*a* = 9.038 (2) Å	θ = 6–20°
*b* = 10.515 (2) Å	µ = 2.48 mm^−^^1^
*c* = 11.869 (2) Å	*T* = 296 K
β = 99.77 (3)°	Plate, colourless
*V* = 1111.6 (4) Å^3^	0.55 × 0.30 × 0.10 mm
*Z* = 4	

### Data collection

**Table:**

Kuma KM-4 diffractometer	1252 reflections with *I* > 2σ(*I*)
Radiation source: fine-focus sealed tube	*R*_int_ = 0.084
graphite	θ_max_ = 67.7°, θ_min_ = 5.0°
ω–2θ scans	*h* = −10→10
Absorption correction: for a cylinder mounted on the φ axis (Dwiggins, 1975)	*k* = −12→12
*T*_min_ = 0.435, *T*_max_ = 0.485	*l* = 0→14
3800 measured reflections	3 standard reflections every 100 reflections
1942 independent reflections	intensity decay: 3.3%

### Refinement

**Table:**

Refinement on *F*^2^	Primary atom site location: structure-invariant direct methods
Least-squares matrix: full	Secondary atom site location: difference Fourier map
*R*[*F*^2^ > 2σ(*F*^2^)] = 0.068	Hydrogen site location: inferred from neighbouring sites
*wR*(*F*^2^) = 0.217	H-atom parameters constrained
*S* = 1.04	*w* = 1/[σ^2^(*F*_o_^2^) + (0.1522*P*)^2^] where *P* = (*F*_o_^2^ + 2*F*_c_^2^)/3
1942 reflections	(Δ/σ)_max_ = 0.013
138 parameters	Δρ_max_ = 0.38 e Å^−^^3^
0 restraints	Δρ_min_ = −0.39 e Å^−^^3^

### Special details

**Table:**

Experimental. cylinder dimensions used for absorption correction: 0.2 mm radius and a 0.1 mm height
Geometry. All s.u.'s (except the s.u. in the dihedral angle between two l.s. planes) are estimated using the full covariance matrix. The cell s.u.'s are taken into account individually in the estimation of s.u.'s in distances, angles and torsion angles; correlations between s.u.'s in cell parameters are only used when they are defined by crystal symmetry. An approximate (isotropic) treatment of cell s.u.'s is used for estimating s.u.'s involving l.s. planes.
Refinement. Refinement of *F*^2^ against ALL reflections. The weighted *R*-factor *wR* and goodness of fit *S* are based on *F*^2^, conventional *R*-factors *R* are based on *F*, with *F* set to zero for negative *F*^2^. The threshold expression of *F*^2^ > 2σ(*F*^2^) is used only for calculating *R*-factors(gt) *etc*. and is not relevant to the choice of reflections for refinement. *R*-factors based on *F*^2^ are statistically about twice as large as those based on *F*, and *R*- factors based on ALL data will be even larger.

### Fractional atomic coordinates and isotropic or equivalent isotropic displacement parameters (Å^2^)

**Table:**

	*x*	*y*	*z*	*U*_iso_*/*U*_eq_	
C1	0.4980 (4)	0.4433 (3)	0.2021 (3)	0.0493 (8)	
C1P	0.3908 (4)	0.4006 (4)	0.3776 (3)	0.0506 (9)	
C2	0.8673 (4)	0.3455 (4)	0.1991 (3)	0.0557 (9)	
C2P	0.3249 (5)	0.5171 (4)	0.3911 (3)	0.0566 (9)	
H2P	0.3381	0.5864	0.3451	0.068*	
C3	0.9760 (5)	0.2575 (6)	0.2680 (4)	0.0785 (14)	
H3A	0.9395	0.2344	0.3366	0.094*	
H3B	1.0715	0.2989	0.2876	0.094*	
H3C	0.9870	0.1823	0.2242	0.094*	
C3P	0.2380 (5)	0.5252 (4)	0.4767 (4)	0.0613 (10)	
C4	0.9221 (5)	0.4267 (5)	0.1128 (4)	0.0718 (13)	
H4A	0.9086	0.3830	0.0408	0.086*	
H4B	1.0268	0.4448	0.1373	0.086*	
H5C	0.8665	0.5049	0.1046	0.086*	
C4P	0.2123 (5)	0.4283 (5)	0.5460 (4)	0.0675 (11)	
H4P	0.1521	0.4384	0.6015	0.081*	
C5P	0.2802 (6)	0.3136 (5)	0.5299 (4)	0.0700 (12)	
H5P	0.2662	0.2447	0.5761	0.084*	
C6P	0.3682 (5)	0.2991 (4)	0.4467 (4)	0.0639 (11)	
H6P	0.4124	0.2209	0.4370	0.077*	
N1	0.4913 (4)	0.3838 (3)	0.3000 (3)	0.0564 (8)	
H1	0.5586	0.3263	0.3189	0.068*	
N2	0.6276 (4)	0.4233 (3)	0.1607 (3)	0.0560 (8)	
H2	0.6427	0.4587	0.0983	0.067*	
N3	0.7337 (4)	0.3444 (4)	0.2220 (3)	0.0567 (8)	
S1	0.36293 (11)	0.53442 (10)	0.12753 (8)	0.0578 (4)	
F1	0.1751 (4)	0.6403 (3)	0.4920 (3)	0.0941 (11)	

### Atomic displacement parameters (Å^2^)

**Table:**

	*U*^11^	*U*^22^	*U*^33^	*U*^12^	*U*^13^	*U*^23^
C1	0.0477 (18)	0.0465 (19)	0.0519 (19)	0.0021 (16)	0.0037 (14)	−0.0012 (15)
C1P	0.0412 (17)	0.061 (2)	0.0499 (18)	0.0004 (16)	0.0070 (14)	0.0020 (16)
C2	0.052 (2)	0.057 (2)	0.058 (2)	0.0001 (18)	0.0065 (17)	−0.0026 (18)
C2P	0.063 (2)	0.052 (2)	0.055 (2)	0.0007 (18)	0.0113 (17)	0.0036 (17)
C3	0.062 (3)	0.099 (3)	0.073 (3)	0.027 (3)	0.006 (2)	0.007 (3)
C3P	0.072 (3)	0.061 (2)	0.0521 (19)	0.012 (2)	0.0145 (18)	−0.0026 (18)
C4	0.049 (2)	0.087 (3)	0.078 (3)	−0.008 (2)	0.0078 (19)	0.011 (3)
C4P	0.071 (3)	0.078 (3)	0.058 (2)	0.008 (2)	0.0247 (19)	0.007 (2)
C5P	0.075 (3)	0.071 (3)	0.068 (3)	0.001 (2)	0.026 (2)	0.016 (2)
C6P	0.068 (3)	0.057 (2)	0.067 (2)	0.006 (2)	0.0116 (19)	0.0073 (19)
N1	0.0545 (18)	0.0620 (19)	0.0541 (17)	0.0156 (16)	0.0133 (13)	0.0080 (15)
N2	0.0498 (17)	0.0609 (18)	0.0572 (18)	0.0055 (15)	0.0087 (13)	0.0070 (15)
N3	0.0502 (17)	0.064 (2)	0.0564 (17)	0.0092 (15)	0.0107 (14)	0.0049 (15)
S1	0.0535 (6)	0.0664 (7)	0.0528 (6)	0.0100 (5)	0.0069 (4)	0.0064 (4)
F1	0.139 (3)	0.0729 (18)	0.0802 (18)	0.0358 (19)	0.0475 (18)	0.0055 (15)

### Geometric parameters (Å, °)

**Table:**

C1—N1	1.331 (5)	C3P—C4P	1.354 (6)
C1—N2	1.361 (5)	C3P—F1	1.362 (5)
C1—S1	1.681 (4)	C4—H4A	0.9600
C1P—C6P	1.382 (6)	C4—H4B	0.9600
C1P—C2P	1.383 (6)	C4—H5C	0.9600
C1P—N1	1.409 (5)	C4P—C5P	1.381 (7)
C2—N3	1.282 (5)	C4P—H4P	0.9300
C2—C4	1.482 (6)	C5P—C6P	1.377 (6)
C2—C3	1.489 (6)	C5P—H5P	0.9300
C2P—C3P	1.389 (6)	C6P—H6P	0.9300
C2P—H2P	0.9300	N1—H1	0.8600
C3—H3A	0.9600	N2—N3	1.379 (5)
C3—H3B	0.9600	N2—H2	0.8600
C3—H3C	0.9600		
			
N1—C1—N2	114.4 (3)	C2—C4—H4B	109.5
N1—C1—S1	126.2 (3)	H4A—C4—H4B	109.5
N2—C1—S1	119.4 (3)	C2—C4—H5C	109.5
C6P—C1P—C2P	120.3 (4)	H4A—C4—H5C	109.5
C6P—C1P—N1	117.9 (4)	H4B—C4—H5C	109.5
C2P—C1P—N1	121.6 (4)	C3P—C4P—C5P	116.6 (4)
N3—C2—C4	126.1 (4)	C3P—C4P—H4P	121.7
N3—C2—C3	115.8 (4)	C5P—C4P—H4P	121.7
C4—C2—C3	118.1 (4)	C6P—C5P—C4P	121.3 (4)
C1P—C2P—C3P	116.6 (4)	C6P—C5P—H5P	119.3
C1P—C2P—H2P	121.7	C4P—C5P—H5P	119.3
C3P—C2P—H2P	121.7	C5P—C6P—C1P	120.1 (4)
C2—C3—H3A	109.5	C5P—C6P—H6P	119.9
C2—C3—H3B	109.5	C1P—C6P—H6P	119.9
H3A—C3—H3B	109.5	C1—N1—C1P	129.9 (3)
C2—C3—H3C	109.5	C1—N1—H1	115.0
H3A—C3—H3C	109.5	C1P—N1—H1	115.0
H3B—C3—H3C	109.5	C1—N2—N3	117.8 (3)
C4P—C3P—F1	118.1 (4)	C1—N2—H2	121.1
C4P—C3P—C2P	125.0 (4)	N3—N2—H2	121.1
F1—C3P—C2P	116.9 (4)	C2—N3—N2	118.6 (4)
C2—C4—H4A	109.5		

### Hydrogen-bond geometry (Å, °)

**Table:**

*D*—H···*A*	*D*—H	H···*A*	*D*···*A*	*D*—H···*A*
N1—H1···N3	0.86	2.12	2.553 (5)	111
N2—H2···S1^i^	0.86	2.67	3.465 (3)	154

Symmetry codes:  (i) −*x*+1, −*y*+1, −*z*.
